# Neuroprotective Effects of *Sparassis crispa* Ethanol Extract through the AKT/NRF2 and ERK/CREB Pathway in Mouse Hippocampal Cells

**DOI:** 10.3390/jof9090910

**Published:** 2023-09-07

**Authors:** Malk Eun Pak, Wei Li

**Affiliations:** Korean Medicine (KM)-Application Center, Korea Institute of Oriental Medicine, Daegu 41062, Republic of Korea; clear46@kiom.re.kr

**Keywords:** *Sparassis crispa*, cauliflower mushroom, neuroprotection, edible mushroom, medicinal mushroom

## Abstract

*Sparassis crispa*, known as the “Cauliflower mushroom”, is an edible medicinal fungus found in Asia, Europe, and North America. Its fruiting bodies contain active biological and pharmacological ingredients with antitumor and anti-inflammatory properties. In this study, we investigated the neuroprotective effect of various *Sparassis crispa* extract against glutamate-induced toxicity and oxidative stress in hippocampal HT22 cells. Cell viability and reactive oxygen species (ROS) analyses served to evaluate the neuroprotective effects of *Sparassis crispa* ethanol extract (SCE) and their fractions partitioned with ethyl acetate (EtOAc; SCE-E) and water (SCE-W) in HT22 cells. SCE and SCE-E treatment reduced glutamate-induced cell death and ROS generation. SCE-E reduced apoptosis and ROS levels by regulating anti-apoptotic proteins. Under glutamate treatment, SCE-E activated nuclear factor erythroid-derived 2-related factor 2 (Nrf2) and regulated extracellular signal-regulated kinase (ERK) and AKT signals at late stages. SCE-E increased the protein expression of cAMP response element binding (CREB), brain-derived neurotrophic factor (BDNF), and Kelch-like ECH-associated protein 1 (Keap1), and decreased the Nrf2 protein expression. Moreover, co-treatment of SCE-E and wortmannin did not activate Nrf2 expression. Thus, the neuroprotective effect of SCE-E is likely due to Nrf2 and CREB activation through AKT and ERK phosphorylation, which effectively suppress glutamate-induced oxidative stress in HT22 cells. Accordingly, a daily supplement of SCE-E could become a potential treatment for oxidative-stress-related neurological diseases.

## 1. Introduction

Medicinal mushrooms have an established history of use in traditional therapies; recently, mushrooms such as *Fomes fomentarius*, *Ganoderma lucidum*, and *Armillaria ostoyae*, have shown potential as pharmaceutical agents [1]. Dietary interventions and food improve cognition throughout life [2], as well as being promising for delaying the symptoms of dementia [3]. Dietary supplement-based strategies have been demonstrated to be effective in subjects with mild cognitive impairment [4], demonstrating that bioactive components from foods or edible fungi are efficient, safe, and accessible.

Oxidative stress results from imbalanced pro-oxidant/antioxidant homeostasis, which leads to the generation of toxic reactive oxygen species (ROS). Constitutive ROS generation is involved in cell death processes in neuronal cells [5] and has been implicated in the progression of neurodegenerative diseases such as Alzheimer’s disease (AD) [6]. Glutamate is an endogenous excitatory neurotransmitter involved in many aspects of brain function, including cognition, memory, and learning [7]. However, the release of excessive amounts of glutamate by neurons after brain injury can induce neuronal apoptosis through two distinct mechanisms—excitotoxicity and oxidative stress [8,9]. Thus, glutamate-induced neurotoxicity results in oxidative stress and cell death in HT22 cells, an immortalized mouse hippocampal cell line [10].

To maintain a proper redox balance, the central nervous system has an antioxidant defense mechanism consisting of endogenous antioxidant enzymes, whose expression is tightly controlled by the antioxidant response element (ARE) and activated by nuclear factor erythroid 2-related factor 2 (NRF2) [11]. NRF2 transcription is repressed by its negative regulator Kelch-like ECH-associated protein 1 (Keap1); however, upon exposure to ROS, Nrf2 dissociates from cytosolic Keap1 and translocates to the nucleus, where it binds to the ARE in the promoter region of genes encoding antioxidant enzymes, thereby inducing the production of endogenous antioxidant enzymes [12]. AKT signaling facilitates NRF2 activation by inhibiting the interaction with Keap1 [13] and promotes the cAMP response element-binding protein (CREB)/brain-derived neurotrophic factor (BDNF) signaling pathway [14]. The BDNF-dependent TrkB pathway is an essential signaling pathway for the survival and normal functioning of mature neurons, which play pivotal roles in memory and cognition [15]. Extracellular signal-regulated kinase (ERK) signaling drives CREB transcription, which can promote synaptic plasticity and BDNF release [16,17]. Previous research has shown that N-acetyl serotonin has neuroprotective effects through the activation of both the CREB/BDNF pathway and AKT/NRF2/antioxidant enzymes in HT22 cells [18]. Therefore, activating BDNF/TrkB and the NRF2-ARE signaling system offer a potential approach to the design of novel therapeutic agents for AD [15].

*Sparassis crispa*, known as “Cauliflower mushroom” or “*Sparassis latifolia*”, is a fungus species belonging to the genus Sparassis that contains highly active biological and pharmacological ingredients (e.g., β-glucan, antifungal compounds (sparassol, methyl-2, 4-dihydroxy-6-methylbenzoate and methyl-dihydroxymethoxy-methylbenzoate), ergosterol peroxides, and benzoate derivatives) [19,20]. Accordingly, *Sparassis crispa* has antitumor, anti-inflammatory, antiviral, antihypertensive, antiallergic, and antidiabetic activities and is used for medicinal purposes [20]. Recently, a study showed that a neutral polysaccharide from *Sparassis crispa* ameliorated H_2_O_2_-induced neurotoxicity in HT22 cells [21]. However, the signals mediating the neuroprotective or antioxidant effects of *Sparassis crispa* in HT22 cells remain unknown.

In our study, we aimed to identify the mechanisms underlying the neuroprotective effects of *Sparassis crispa* ethanol extract (SCE) and its fractions (ethyl acetate, EtOAc, and water) against glutamate-induced oxidative stress. In addition, we studied those EtOAc fractions (SCE-E) of *Sparassis crispa* that showed antioxidant properties via regulating AKT/NRF2 and ERK/CREB signaling in glutamate-induced toxicity.

## 2. Materials and Methods

### 2.1. Fungal Material

Dried fruiting bodies of *Sparassis crispa* were purchased from a herbal market in Seoul, Korea, in August 2020. The scientific name of the specimens was confirmed by one of authors (Wei Li). *Sparassis crispa* was deposited as a voucher specimen (mushroom no.2, M2) at the Korean Medicine-Application Center, Korea Institute of Oriental Medicine.

### 2.2. Extraction and Separation

**Figure 1:**
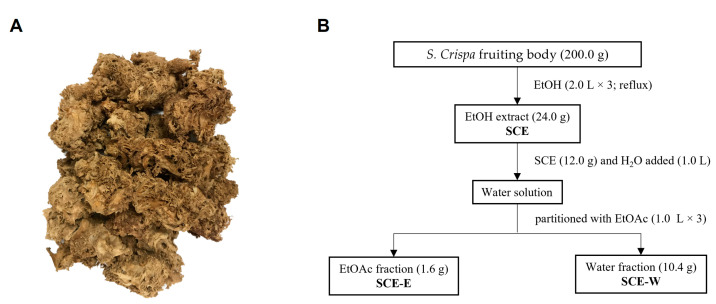
*Sparassis crispa* material and sample preparation of *Sparassis crispa*. (**A**) Dried fruiting body and (**B**) extraction and separation scheme of *Sparassis crispa*. SCE: *Sparassis crispa* ethanol extract; SCE-E: SCE EtOAc fraction; SCE-W: SCE water fraction; EtOH: ethanol; EtOAc: ethyl acetate.

Dried fruiting bodies (200.0 g) of *Sparassis crispa* (Figure 1A) were extracted with EtOH (2 L × 3) under reflux. Half of the ethanol (EtOH) extract (SCE; 12.0 g) was suspended in water and partitioned with EtOAc, yielding EtOAc (SCE-E; 1.6 g) and water (SCE-W; 10.4 g) fractions (Figure 1B).

### 2.3. Cell Culture

The mouse hippocampus cell line, derived from HT22 cells, was cultured in Dulbecco′s Modified Eagle′s Medium (DMEM, Hyclone) supplemented with 10% fetal bovine serum (FBS) and 1% penicillin/streptomycin (Gibco-Invitrogen, Carlsbad, CA, USA) in 5% CO_2_ at 37 °C. The cells were cultured in a 96-well plate or 6-well plate and incubated for 24 h. After incubation with various concentrations of SCE, SCE-W, and SCE-E for 24 h, the cells were treated by co-treatment with 5 mM glutamate and extracts for 24 h or as part of a time-dependent experiment.

### 2.4. Cell Viability Assay

For determination of the cell viability, after all treatments, 3-(4,5-Dimethylthiazol-2-yl)-2,5-Diphenyltetrazolium Bromide (MTT) solution was added in the culture media. Following incubation in the dark for 2 h at 37 °C, the absorbance was determined at 540 nm using Spetra-Max i3 (Molecular devices, Sunnyvale, CA, USA). The results are expressed as a percentage of the control cells.

### 2.5. Annexin Ⅴ/Propidium Iodide (PI) Assay

For determination of the cell apoptotic cell death, flow cytometric analysis was performed using the fluorescein 5(6)-isothiocyanate (FITC) Annexin V/PI apoptosis detection kit (BD Pharmingen^TM^, San Diego, CA, USA). The HT22 cells were harvested and washed with PBS. Then, 5% Annexin Ⅴ and 5% PI solution was added to each sample and incubated for 20 min. The samples were analyzed using a CytoFLEX flow cytometer (Beckman Coulter, Brea, CA, USA).

### 2.6. Intracellular ROS Measurement

For the determination of ROS using a microplate, adherent cells were added to 96-well plates stained with 20 μM carbox-H_2_DCFDA (Sigma) in a medium for 45 min, and then washed in PBS. The plates were analyzed with a microplate reader at 485 nm/535 nm. In flow cytometry, the cells were harvested after treatment, washed with PBS, and incubated with carbox-H_2_DCFDA (Sigma) for 60 min in darkness. These samples were analyzed using a flow cytometer (Beckman Coulter). In fluorescent microscopy, adherent cells in 6-well plates were washed with PBS and stained carbox-H_2_DCFDA for 45 min. Then, the cells were washed in PBS and analyzed with a microscope (Olympus).

### 2.7. Real-Time Quantitative Reverse Transcription Polymerase Chain Reaction (RT-qPCR)

The total RNA in HT22 cells was isolated using the RNeasy Mini, RNA isolation kit (Qiagen, Chatsworth, CA, USA) according to the manufacturer’s protocol. Extracted RNA was synthesized into cDNA using Omniscript Reverse Transcriptase (Qiagen). SYBR green-based quantitative PCR amplification was performed using the QuantStudio 6 Flex Real-time PCR System (Thermo Scientific, Waltham, MA, USA). The relative quantitation values of the respective mRNAs were normalized to that of the endogenous β-actin control and were quantified by the 2^–ΔΔCt^ method. The set of primers were used to amplify the mouse specific products (Supplemental Table S1).

### 2.8. Western Blot

The cells were homogenized with a radioimmunoprecipitation assay (RIPA) lysis buffer and incubated for 20 min. Then, they were centrifuged at 12,000 rpm in 4 °C for 20 min, and the supernatant was collected. A purified test protein (BSA 2 mg/mL) was diluted into 2, 4, 6, 8, and 10 µg/mL, which was used to construct a standard curve using a protein assay kit (#23227, Thermo Scientific). Protein cencertration of sample were calculated from the curve. The samples were loaded onto 10% sodium dodecyl sulfate polyacrylamide gel electrophoresis (SDS-PAGE) gel and then transferred to a polyvinylidene difluoride (PVDF) membrane. The membranes were blocked for 3% bovine serum albumin (BSA) solution and incubated with following primary antibodies in 5% BSA solution: anti-β-actin (sc-47778), Bcl-2 (#3498), Bax (#2772), HO-1 (#82206), NQO-1 (sc-32793), Nrf2 (NBP1-32822), Keap1 (#8047), pERK (#9101), ERK (#9102), pAKT (#9101), AKT (#9102), pCREB (#9198), and BDNF (NBP100-98682) at 4 °C overnight. The membranes were washed with tris-buffered saline with tween 20 (TBST) and incubated with HRP-conjugated secondary antibodies (A90-137P or A120-108P) for 1 h at room temperature. Then, the membranes reacted with an ECL solution (Pierce, Rockford, IL, USA) and immunoreactivity was recorded using a digital imaging system (Q9 Alliance; UVITEC Ltd., Cambridge, England, UK). The results were quantified using ImageJ 1.52v software and normalized to β-actin.

### 2.9. High Performance Liquid Chromatography (HPLC) Analysis

All standard solutions for HPLC analysis were dissolved in methanol. Standard stock solutions of the compounds were prepared at a concentration of 1 mg/mL and were then serially diluted with methanol to obtain a calibration curve of standard solutions. SCE-E was prepared at a concentration of 5 mg/mL. All of the solutions for analysis were filtered through 0.45 μm regenerated cellulose membrane syringe filters (Sartorius, Göttingen, Germany). HPLC analysis was performed using a Dionex UltiMate 3000 system (Dionex Corp., Sunnyvale, CA, USA) equipped with a binary pump, autosampler, column oven, and diode array UV/VIS detector. Separation was performed on a Luna C18 column (250 × 4.6 mm, 5 μm, Phenomenex, Torrance, CA, USA), with the column oven temperature remaining at 30 °C, at an ultraviolet wavelength of 254 nm. The mobile phase consisted of water (solvent A) and acetonitrile (solvent B) with a gradient elution of 65 min, 5% B (0–5 min), 5–50% B (0–20 min), 5–60% B (5–20 min), and 60–100% B (20–50 min), 100% B (50–65 min) at a flow rate of 1.0 mL/min. Before injection of the next sample, the column was re-equilibrated with the initial gradient of solvents for 10 min.

### 2.10. Statstical Analysis

All data were expressed as mean ± standard error of the mean (SEM) and were analyzed using PRISM5 (GraphPad Software, San Diego, CA, USA). Statistical comparisons of more than two groups were performed by one-way analysis of variance, followed by Tukey’s post hoc test. A *p* value of 0.05 was considered statistically significant.

## 3. Results

### 3.1. Effects of SCE on Glutamate-induced Excitotoxicity and ROS Generation in HT22 Cells

**Figure 2:**
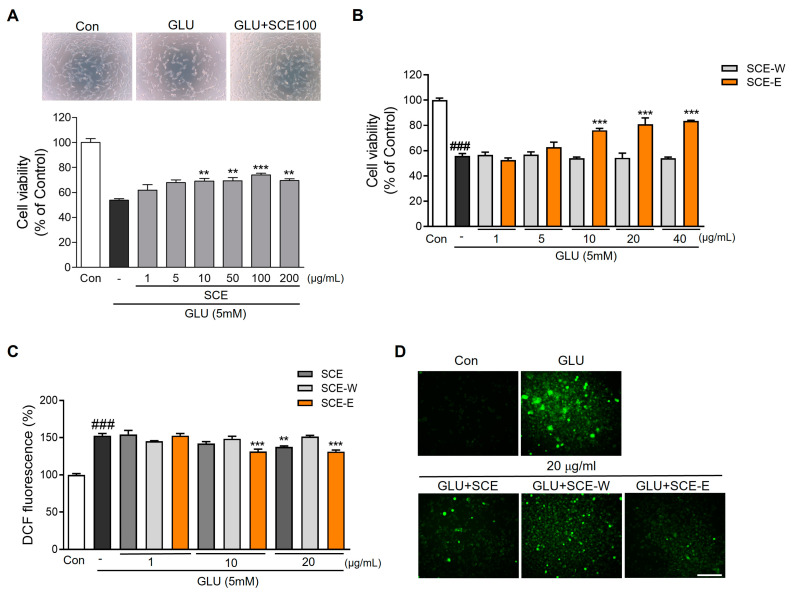
Effects of *Sparassis crispa* ethanol extract and fractions on glutamate-induced cell death and ROS production. (**A**) Cell viability assay and morphological changes after SCE with glutamate treatment. (**B**) Cell viability assay after SCE-E and SCE-W with glutamate treatment. (**C**) Intracellular ROS levels were detected and (**D**) photomicrographs using carboxy-H_2_DCFDA after SCE, SCE-E, and SCE-W with glutamate treatment. SCE significantly enhanced cell survival dose dependently, and specially SCE-E reduced cell death and ROS production. All data are expressed as the mean ± SEM. ^###^ *p* < 0.001 vs. Con; ** *p* < 0.01, *** *p* < 0.001 vs. GLU. N = 6. Scale bar = 100 μm. Con: Control; GLU: glutamate; SCE: *Sparassis crispa* ethanol extract; SCE-E: SCE EtOAc fraction; SCE-W: SCE water fraction; ROS: intracellular reactive oxygen species; -: glutamate treatment cells

We investigated the protective effect of various SCEs by measuring cell viability. First, we tested whether SCE has toxicity and found no toxicity of SCE extracts (1~400 µg/mL). SCE extracts (10–200 µg/mL) significantly increased cell viability after glutamate-induced excitotoxicity (Figure 2A). SCE was separated by solvent polarity (EtOAc and water) using a separatory funnel to obtain two fractions: SCE-E and SCE-W. SCE-E and SCE-W had no toxicity in a 0.1~20 µg/mL dose (Supplemental Figure S1), and a 10–50 μg/mL SCE-E extract but not a SCE-W extract, increased cell viability under glutamate-induced excitotoxicity in a dose-dependent manner (Figure 2B). Next, we measured ROS generation using DCFDA after SCE, SCE-E, and SCE-W treatment. Glutamate treatment increased ROS accumulation, while 10–20 μg/mL SCE-E and 20 μg/mL SCE treatments reduced it significantly (Figure 2C,D). These results indicate that SCE has neuroprotective effects on glutamate-induced excitotoxicity and against oxidative stress, and among them, SCE-E is related to these effects.

### 3.2. Protective Effect of SCE-E against Glutamate-Induced Cell Death in HT22 Cells

**Figure 3:**
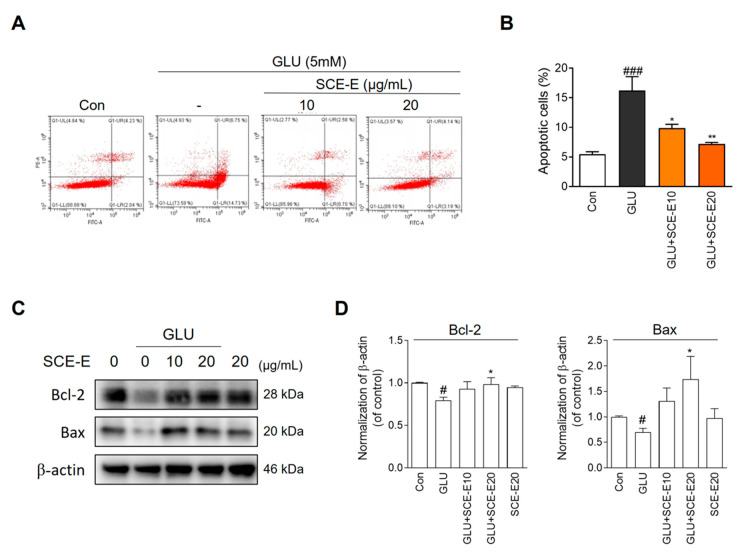
Effects of SCE-E on glutamate-induced apoptotic cell death. (**A**) Representative flow cytometry analysis scatter-gram and (**B**) histogram for Annexin Ⅴ/PI staining. (**C**) Western blot and (**D**) its histogram for Bcl-2 and Bax. All data are expressed as the mean ± SEM. ^#^ *p* < 0.05, ^###^ *p* < 0.001 vs. Con; * *p* < 0.05, ** *p* < 0.01 vs. GLU. N = 6 or 3. Con: Control; GLU: glutamate; SCE-E: SCE EtOAc fraction.

As the 20 μg/mL SCE-E dose was the most effective, we chose two doses (10 and 20 μg/mL) for further experiments. In flow cytometry using annexin V/PI, glutamate treatment significantly increased the apoptosis rate compared with the control, whereas 10 and 20 μg/mL SCE-E treatment reduced the glutamate-induced apoptosis rate (Figure 3A,B). Next, we measured Bcl-2 and Bax protein expression by Western blot (Figure 3C). Under glutamate treatment, Bcl-2 expression increased with SCE-E treatment. In addition, glutamate treatment decreased the expression of Bax, while SCE-E treatment increased it (Figure 3D). These results indicated that SCE-E treatment can reduce glutamate-induced apoptosis in HT22 cells.

### 3.3. Protective Effect of SCE-E against Glutamate-Induced Oxidative Stress in HT22 Cells

**Figure 4:**
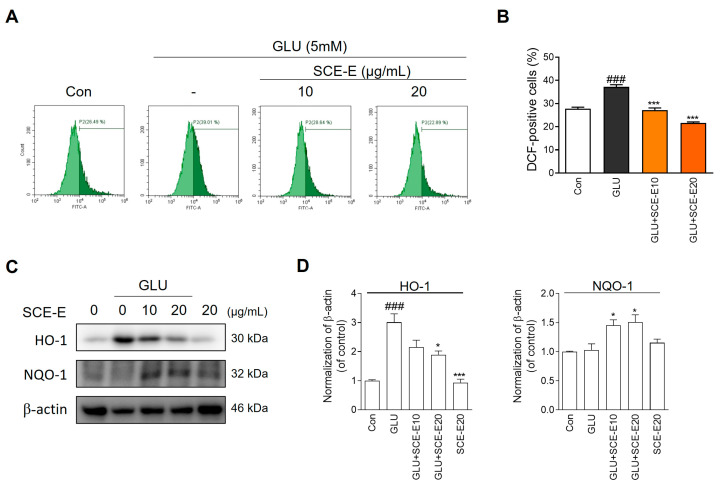
Effects of SCE-E on glutamate-induced ROS production. Representative flow cytometry analysis for (**A**) carboxy-H_2_DCFDA and (**B**) it’s histograms. (**C**) Western blot and (**D**) its histogram for HO-1 and NQO-1. All data are expressed as the mean ± SEM. ^###^ *p* < 0.001 vs. Con; * *p* < 0.05, *** *p* < 0.001 vs. GLU. N = 3. Con: Control; GLU: glutamate; SCE-E: SCE EtOAc fraction; ROS: intracellular reactive oxygen species; HO-1: heme oxygenase-1; NQO1: NAD[P]H: quinone oxidoreductase 1.

To investigate the effect of SCE-E on glutamate-induced oxidative stress, we performed DCFDA staining using flow cytometry. SCE-E (10 and 20 μg/mL) treatment significantly reduced ROS accumulation compared with glutamate treatment alone (Figure 4A,B). To confirm these effects, we measured HO-1 and NQO1 protein expression using Western blot. SCE-E treatment decreased the elevated expression of HO-1 in glutamate treated cells. In addition, SCE-E treatment increased the decreased NQO-1 expression caused by glutamate treatment (Figure 4C,D). These results indicated that SCE-E treatment can reduce glutamate-induced ROS generation in HT22 cells.

### 3.4. SCE-E Regulates Gene Expression of Bdnf, Nrf2, and Catalase in HT22 Cells

**Figure 5:**
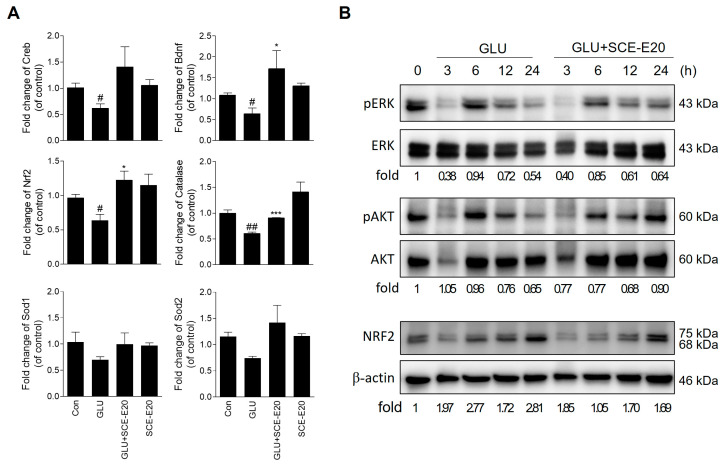
Effects of SCE-E on expression of antioxidant-related gene and phosphorylation of ERK/AKT signaling in glutamate-induced oxidative stress. (**A**) Gene expression of Creb, Bdnf, Nrf2, catalase, Sod1, and Sod2. (**B**) Western blot for pERK, ERK, pAKT, AKT, and Nrf2 in time-course in glutamate and SCE-E-treated cells. Data for gene expression are expressed as the mean ± SEM. ^#^ *p* < 0.05, ^##^ *p* < 0.01 vs. Con; * *p* < 0.05, *** *p* < 0.001 vs. Glutamate. N = 3. Con: Control; GLU: glutamate; SCE-E: SCE EtOAc fraction; ERK: extracellular signal-regulated kinase; pERK: phosphorylated ERK; pAKT: phosphorylated AKT; Nrf2: nuclear factor erythroid-2-related factor 2.

Next, we investigated the effects of SCE-E over the expression of antioxidant and neuroprotection-related genes. Glutamate treatment reduced CREB and BDNF expression compared with the controls, but SCE-E significantly increased the expression of these genes compared with glutamate treatment alone. Furthermore, glutamate treatment downregulated Nrf2, catalase, Sod1, and Sod2 expression compared with the controls, which is being upregulated by SCE-E treatment (Figure 5A). These results indicated that SCE-E treatment can regulate the expression of antioxidant genes and CREB-BDNF under glutamate treatment.

### 3.5. SCE-E Regulates ERK-Mediated CREB-BDNF Signaling and AKT-Mediated Nrf2 Signaling

**Figure 6:**
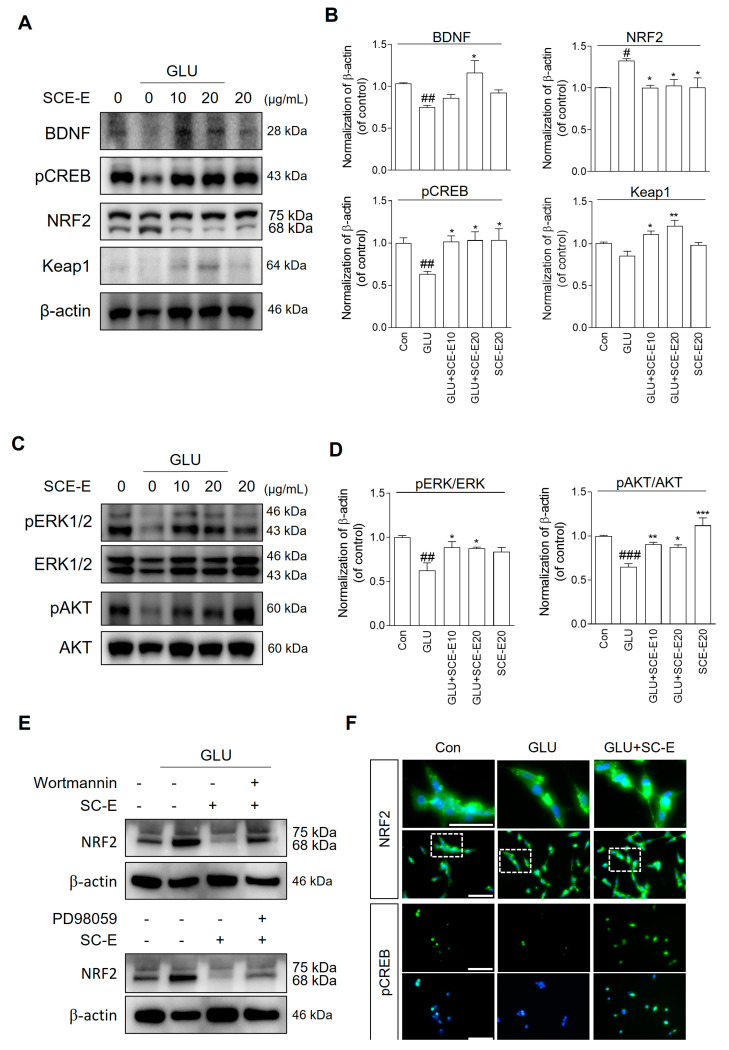
SCE-E regulates ERK/CREB and AKT/NRF2 signaling in glutamate-induced oxidative stress. (**A**) Western blot and its histogram for (**B**) BDNF, pCREB, NRF2, and Keap1. (**C**) Protein expression and (**D**) its densitometric histogram for pERK/ERK and pAKT/AKT using Western blot. (**E**) Expression of NRF2 protein by SCE-E in HT22 cells treated with glutamate with/without wortmannin or PD98059 and (**F**) its representative photographs. All data are expressed as the mean ± SEM. ^#^ *p* < 0.05, ^##^ *p* < 0.01, ^###^ *p* < 0.001 vs. Con; * *p* < 0.05, ** *p* < 0.01, *** *p* < 0.001 vs. GLU. N = 3. Con: Control; GLU: glutamate; SCE-E: SCE EtOAc fraction. BDNF: brain-derived neurotrophic factor; pCREB: phosphorylated cAMP response element binding protein; Nrf2: nuclear factor erythroid-2-related factor 2; Keap1: Kelch-like ECH-associated protein 1, ERK: extracellular signal-regulated kinase; pERK: phosphorylated ERK; pAKT: phosphorylated AKT.

To better understand the mechanisms underlying the observed effects of SCE-E, we investigated whether SCE-E regulates ERK, AKT, and NRF expression (Figure 5B). SCE-E treatment, but not glutamate, increased ERK and AKT levels in 24 h. NRF2 expression in the total cell increased in 3 and 24 h with glutamate treatment, being reduced by SCE-E treatment for 3–24 h. These results indicate that, under glutamate treatment, the antioxidant effect of SCE-E was related to ERK and AKT signaling in the late stages. To confirm that the neuroprotective effect of SCE-E occurred by ERK and AKT upstream, we investigated the protein level related to these (Figure 6A). Glutamate treatment decreased BDNF and pCREB levels as well as Keap1, while SCE-E treatment significantly increased their levels (Figure 6B). In contrast, NRF2 levels increased with glutamate treatment, decreasing with SCE-E treatment. In addition, SCE-E significantly increased the lower pERK/ERK and pAKT/AKT level resulting from glutamate treatment (Figure 6C,D). To investigate whether SCE-E treatment activated NRF2 through both ERK and AKT signaling, we investigated NRF2 expression using wortmannin and PD98059. SCE-E treatment together with wortmannin did not decrease NRF2 expression, but together with PD98059, Nrf2 levels decreased under glutamate treatment (Figure 6E,F). These results indicate that SCE-E treatment can regulate ERK/CREB/BDNF and AKT/NRF2-Keap1 signaling under glutamate treatment.

### 3.6. HPLC Analysis

**Figure 7:**
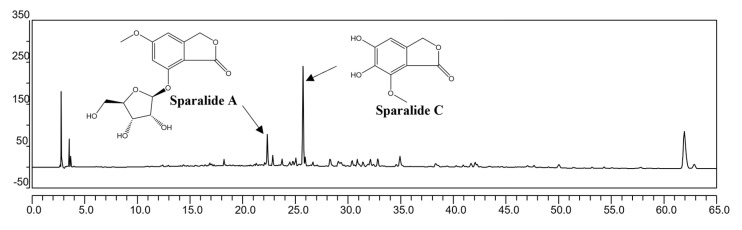
HPLC chromatogram of SCE-E at UV wavelengths of 254 nm.

In our study, we employed HPLC analysis to identify the principal components of SCE-E. The results revealed that sparalides A and C were the main compounds (Figure 7). Our previous research extensively explored various small-molecule compounds present in *Sparassis Crispa*, encompassing aromatic compounds such as sparalides A-C, sparoside A, hanabiratakelide A, 5-hydroxy-7-methoxyphthalide, and 5-methoxy-7-hydroxyphthalide, as well as amino-acid-related compounds (nicotinamide, 5′-deoxy-5′-methylthioadenosine, adenosine) and sterols (ergosterol, ergosterol peroxide, and 5α,6α-epoxy-(22E,24R)-ergosta-8(14),22-diene-3β,7β-diol) [22]. Notably, sparalides A and C emerged as the primary constituents. Moving forward, our research will concentrate on investigating the neuroprotective effects of these key small-molecule compounds, aiming to identify the specific compound responsible for the observed neuroprotective effects.

## 4. Discussion

*Sparassis crispa* is a well-known medical and edible fungus and contains various bioactive and pharmacological compounds, including benzoate derivatives, sesquiterpenoids, maleic acid derivatives, and polysaccharides [22]. *Sparassis crispa* polysaccharides and small molecular explained polysaccharide fraction (SCE-W), but the small-molecule fraction was represented by SCE-E. Recent research reported that crude polysaccharides from SCE exhibited a good antioxidant capacity and could protect HT22 cells against H_2_O_2_-induced oxidative injury [21,23]. In our system, the small-molecule fraction (SCE-E), not the polysaccharide fraction (SCE-W), was the one with neuroprotective effects. However, antioxidant effects of SCE-W through the molecular mechanism have not been investigated against oxidative stress-induced damage in neuronal cells.

Oxidative stress is a contributing factor in the progression of AD. Moreover, increasing ROS by loss of mitochondrial function and reduced antioxidant defenses directly affect synaptic activity and neurotransmission, leading to cognitive dysfunction [24]. Glutamate cytotoxicity is associated with ROS generation through nonreceptor-mediated oxidative toxicity in HT22 cells [25]. Recently, the prevention of glutamate-mediated neurotoxicity has been a therapeutic target in AD [26]. SCE and SCE-E reduced cell damage and oxidative stress under glutamate exposure (Figure 2, Figure 3 and Figure 4). In all experiments, SCE-E was more effective than SCE, suggesting that SCE-E is the fraction with neuroprotective properties.

The Keap1-Nrf2 system regulates the transcription of numerous antioxidant enzymes, such as SOD, CAT, HO-1, glutathione reductase, and NQO1 [27,28]. Simultaneously, Keap1-Nrf2 signaling is modulated by a more complex regulatory network, including phosphoinositide 3-kinase (PI3K)/AKT, ERK and mitogen-activated protein kinase, and protein kinase C [29]. Interestingly, the ERK signaling pathway has a neuroprotective role against glutamate-induced excitotoxicity in hippocampal neurons [30]. Similarly, ERK-CREB signaling has neuroprotective functions [31] and mediates hippocampal-dependent learning triggered by BDNF [17,32]. Previous research has shown that various natural products could serve as potential treatment for AD by attenuating oxidative stress through AKT-mediated NRF2 and ERK-CREB signaling [14,15,33]; similarly, SCE-E has antioxidant effects mediated by these signaling cascades (Figure 6).

Interestingly, SCE-E treatment together with a PI3K inhibitor, wortmannin, did not translocate NRF2, not with the ERK inhibitor, PD-98059. This suggests that NRF2 activation by SCE-E is mediated by PI3K/AKT signaling (Figure 6F). In addition, NRF2 activation by SCE-E treatment occurs at the early stage (~6 h), while pAKT expression decreases at 3–12 h compared with glutamate treatment alone. In addition, SCE-E treatment alone induced Cat and NRF2 gene mimic, not Sod2 and CREB (Figure 5). Thus, it appears that SCE-E activates NRF2 in the early stage through another signaling cascade. However, we did not investigate this point, which is a limitation of our study.

In our data, HO-1 expression induced by glutamate exposure was reduced by SCE-E treatment (Figure 4C). HO-1, an effective ROS scavenger, is inducible following exposure to hydrogen peroxide [34]; thus, HO-1 expression is a marker of ROS accumulation. Mitochondrial SOD2, but not cytosolic SOD1, in the nervous system may represent an effective therapeutic strategy against oxidative stress-induced neuronal death [35]. In our experiments, SCE-E induced SOD2 expression, not SOD1 (Figure 5A). Previous research indicated that the SCE polysaccharide at a high dose (100–200 µg/mL) protects HT22 cells against oxidative injury [21]; however, the polysaccharide fraction (SCE-W) showed no such effects in our settings. We found the small-molecule fraction (SCE-E) was more effective than large molecule fraction (SCE-W) at a low dose, suggesting that the antioxidant effect of *Sparassis crispa* was exerted by SCE-E from a pharmacological point of view. Possibly the small-molecule constituent of *Sparassis crispa* acts PI3K/AKT signaling directly and ERK signaling indirectly, which show molecular mechanisms acting as a new medicinal mushroom.

## 5. Conclusions

**Figure 8:**
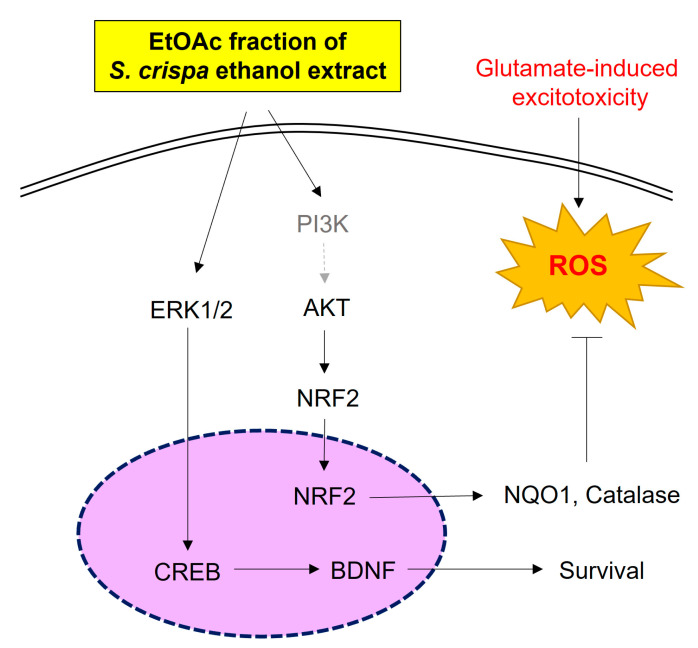
Schema of a hypothesized mechanism by neuroprotective effect of SCE-E in glutamate-induced hippocampal cells. SCE-E: SCE EtOAc fraction; ERK: extracellular signal-regulated kinase; CREB: ERK: extracellular signal-regulated kinase; NRF2: Nuclear factor erythroid-2-related factor 2; Keap1: Kelch-like ECH-associated protein 1; NQO1: NAD[P]H: quinone oxidoreductase 1; SOD: Superoxide Dismutase; ROS: intracellular reactive oxygen species.

In conclusion, SCE and an SCE-E extract can protect cells from glutamate-induced oxidative stress by reducing apoptosis and ROS accumulation. In particular, SCE-E could induce the expression of antioxidant enzymes such as CAT and NQO-1 by activating AKT-NRF2 signaling. In addition, SCE-E increased BDNF expression via ERK-CREB signaling (Figure 8). The present findings suggest that SCE-E may have pharmaceutical applications in AD and other neurodegenerative diseases. In addition, dietary supplementation of SCE-E may protect memory impairment in adults with aging or early neurodegenerative disease.

## Data Availability

All the data are available within the article and Supplementary Materials.

## Supplementary Materials

The following supporting information can be downloaded at: https://www.mdpi.com/article/10.3390/jof9090910/s1, Supplemental Table S1. Primer list; Supplementary Figure S1. Effects of various SCE extracts in HT22 cells on normal condition.
