# Supplementary material for: Neuroprotective Effects of Sparassis crispa Ethanol Extract through the AKT/NRF2 and ERK/CREB Pathway in Mouse Hippocampal Cells
Source: J Fungi (Basel). 2023 Sep 7;9(9):910. doi: 10.3390/jof9090910 (PMC10532724; doi:10.3390/jof9090910)
Supplement: Supplementary file 1 [file jof-09-00910-s001.zip › jof-2551583-supplementary.pdf]

Supplementary materials & data

RT-qRCR

The set of primers were used to amplify mouse specific products below to:

Supplemental table 1. Primer list

Gene	Primer	Sequence
Creb	Forward	5′-TACCCAGGGAGGAGCAATAC-3′
	Reverse	5′-GAGGCAGCTTGAACAACAAC-3′
Bdnf	Forward	5′- CGA CAT CAC TGG CTG ACA CT -3′
	Reverse	5′- CAA GTC CGC GTC CTT ATG GT -3′
Nrf2	Forward	5′- CAGCATAGAGCAGGACATGGAG -3′
	Reverse	5′- GAACAGCGGTAGTATCAGCCAG -3′
Catalase	Forward	5′- CAGCGACCAGATGAAGCA -3′
	Reverse	5′- CTCCGGTGGTCAGGACAT -3′
Sod1	Forward	5′- CAGGACCTCATTTTAATCCTCAC -3′
	Reverse	5′- CCCAGGTCTCCAACATGC -3′
Sod2	Forward	5′- CTGGACAAACCTGAGCCCTA -3′
	Reverse	5′- TGATAGCCTCCAGCAACTCTC -3′
β-actin	Forward	5′-GGCACCACACCTTCTACAATGA-3′
	Reverse	5′-ATCTTTTCACGGTTGGCCTTAG-3′

Cell viability

HT22 cells were cultured at 3 × 10^3 cells/well in 96-well plate and incubated for 24 h. After incubation with various concentration of SCE extractst for 24 h. For determination of cell viability, the CCK assay was used. After treatment, 10% CCK solution was changed into the plate and incubated in the dark for 2 h at 37℃. The absorbance was determined at 450 nm using a microplate reader (Spetra-Max i3, Molecular devices, Sunnyvale, CA, USA). Results are expressed as a percentage of the control cells.

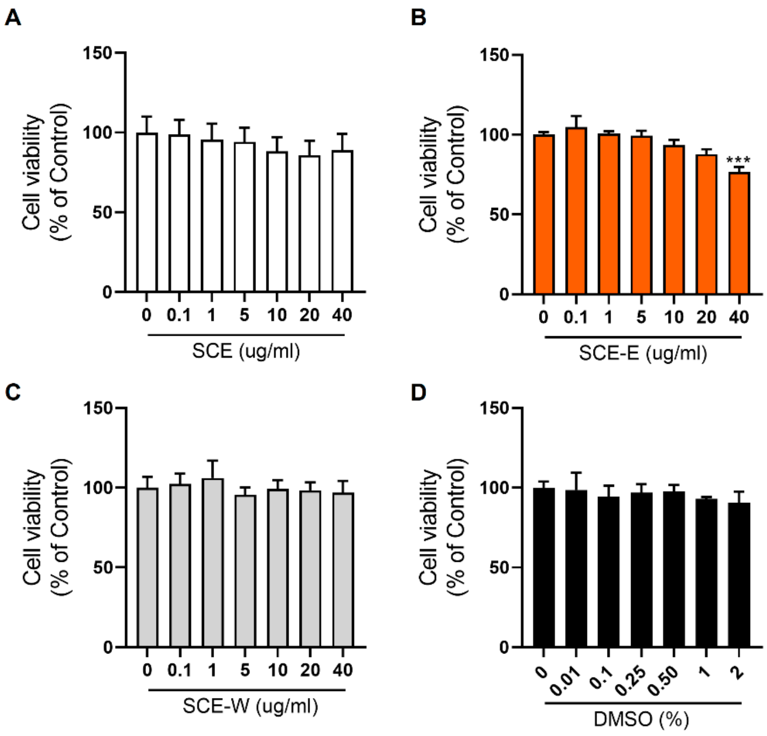

Supplementary Figure 1. Effects of various SCE extracts in HT22 cells on normal condition. Cell viability assay after various dose treatment of (A) SCE, (B) SCE-E and (C) SCE-W extracts without glutamate incubation. (D) Cell viability of various dose treatment of DMSO (solvent of extracts for treatment *in vitro*). In normal condition, various SCE extract does not have toxicity, except for the SCE-E 40ug/ml treatment. All data are expressed as the mean ± SEM. ***P < 0.01 vs. 0 (Control). N = 6.
